# Infectious exposure in the first years of life and risk of central nervous system tumours in children: analysis of birth order, childcare attendance and seasonality of birth

**DOI:** 10.1038/sj.bjc.6605679

**Published:** 2010-05-11

**Authors:** L S Schmidt, M Kamper-Jørgensen, K Schmiegelow, C Johansen, P Lähteenmäki, C Träger, T Stokland, K Grell, G Gustafson, P Kogner, A Sehested, J Schüz

**Affiliations:** 1 Institute of Cancer Epidemiology, Danish Cancer Society, Strandboulevarden 49, DK-2100 Copenhagen, Denmark; 2 Department of Epidemiology Research, Statens Serum Institut, Copenhagen, Denmark; 3 Department of Paediatrics, Copenhagen University Hospital, Copenhagen, Denmark; 4 Department of Obstetrics and Paediatrics, The Medical Faculty, Institute of Gynaecology, University of Copenhagen, Copenhagen, Denmark; 5 Department of Paediatrics, Turku University Hospital, Turku, Finland; 6 Childhood Cancer Research Unit, Department of Woman and Child Health, Karolinska Institutet, Stockholm, Sweden; 7 Department of Paediatrics, University Hospital of North Norway, Tromsø, Norway

**Keywords:** birth order, CNS tumour, childhood cancer, seasonal variation, childcare, infectious disease

## Abstract

**Background:**

An infective, mostly viral basis has been found in different human cancers. To test the hypothesis of a possible infectious aetiology for central nervous system (CNS) tumours in children, we investigated the associations with proxy measures of exposure to infectious disease.

**Methods:**

In a large case–control study nested in the populations of Denmark, Norway, Sweden, and Finland of 4.4 million children, we studied the association of birth order and seasonal variation of birth with subsequent risk for CNS tumours. We identified 3983 children from the national cancer registries, and information on exposure was obtained from the high-quality national administrative health registries. We investigated the association between childcare attendance during the first 2 years of life and the risk for CNS tumours in a subset of Danish children with CNS tumours, using information from the Danish Childcare database.

**Results:**

We observed no association between birth order and risk of CNS tumours overall (odds ratio (OR) for second born or later born *vs* first born, 1.03; 95% confidence interval (CI), 0.96–1.10) or by histological subgroup, and children with CNS tumours did not show a seasonal variation of birth that was distinct from that of the background population. Childcare attendance compared with homecare showed a slightly increased OR (1.29; 95% CI, 0.90–1.86) for CNS tumours, with the highest risk observed in children attending a crèche. The strongest association was observed for embryonal CNS tumours. We found no effect of age at enrolment or duration of enrolment in childcare.

**Conclusion:**

These results do not support the hypothesis that the burden of exposure to infectious disease in early childhood has an important role in the aetiology of paediatric CNS tumours.

Knowledge about the aetiology of central nervous system (CNS) tumours in children is sparse; however, several studies indicate that the pathogenic process of CNS tumours in children is initiated prenatally (Linet *et al*, 1996; Fear *et al*, 2001; Harder *et al*, 2008; Schmidt *et al*, 2010). Furthermore, a few studies indicate that exposure to infectious agents around the time of birth or in early childhood might modulate the risk for CNS tumours (Linet *et al*, 1996; Linos *et al*, 1998; Fear *et al*, 2001; McNally *et al*, 2002; Hoffman *et al*, 2007).

Investigating the role of infectious disease in epidemiological studies presents several challenges. Many infections are subclinical, retrospective recall of an infectious episode is difficult, and serological confirmation is not feasible in large epidemiological studies, particularly when no specific agent is suspected. Alternatively, proxy measures such as birth order, seasonality of birth, and childcare attendance may be used to study the association. An infectious aetiology of CNS tumours is supported by ecological studies of space–time clustering (McNally *et al*, 2002) and seasonal variation of births, with an excess of patients with different subgroups of CNS tumours being born during winter and fall (Yamakawa *et al*, 1979; Hoffman *et al*, 2007; Schmidt *et al*, 2009), although results are inconclusive. Further observational studies indicate that infection during pregnancy (Linos *et al*, 1998; Fear *et al*, 2001; Dickinson *et al*, 2002) or during the neonatal period (Linet *et al*, 1996) might be associated with an increased risk for CNS tumours; however, evidence is limited and results are inconsistent (McKinney *et al*, 1999).

The hypothesis that delayed exposure to common infectious diseases predisposes to CNS tumours was evaluated in two studies, which reported weak protective effects of childcare attendance and social contacts (Shaw *et al*, 2006; Harding *et al*, 2009). The results for the effect of birth order are inconsistent (Linet *et al*, 1996; McCredie *et al*, 1999; Von and Reynolds, 2003), with some studies showing an increased risk among first born (Emerson *et al*, 1991; Linet *et al*, 1996), whereas some show an increased risk in later born (McCredie *et al*, 1999) and other studies show no association (McKinney *et al*, 1999; Schuz *et al*, 2001; Von and Reynolds, 2003).

We conducted a case–control study in a cohort of the Nordic childhood population of ∼4.4 million 0- to 14-year-old children annually, to investigate infectious disease in relation to the risk for developing a CNS tumour, using birth order and season of birth as proxy measures. Furthermore, in a subset of the data, we studied the effect of early childcare attendance on the risk of CNS tumours in Denmark.

## Methods

All residents of the Nordic countries are assigned a unique personal identification number that includes information on sex and date of birth. This ensures unambiguous record linkage between the nationwide administrative registries. In a Nordic case–control study, we identified 3983 children aged 0–14 years in whom a primary CNS tumour had been diagnosed in the period 1985–2006 inclusively, who had resided in Denmark, Norway, Sweden, or Finland at the time of diagnosis (Table 1). Patients were identified from the national cancer registries (Tulinius *et al*, 1992). In addition, in Sweden and Denmark, cases were ascertained from childhood cancer registries and, for Norwegian patients, from the solid-tumour database of the Nordic Society of Paediatric Haematology and Oncology.

Diagnostic information of the Swedish (Lannering *et al*, 2009) and most of the Danish cases (Thorsteinsson *et al*, 2005; Raaschou-Nielsen *et al*, 2006) was reabstracted and recoded on the basis of information from individual medical records. In Finland, pathology reports were consulted to classify glia cell tumours more specifically than the data available from the Finnish Cancer Registry. CNS tumours were defined according to main group III of the 3rd edition of the International Classification of Childhood Cancer (Steliarova-Foucher *et al*, 2005), which includes both benign and malignant primary tumours of the CNS, but excludes germ cell tumours and lymphomas located in the CNS.

The study bases available for addressing the hypotheses of associations with birth order, season of birth, or childcare attendance differ, as explained below:

With regard to birth order analyses, each case was matched individually by age (birth month and year), sex, and country to five controls, identified randomly from the Nordic childhood population. Controls were identified from the national population registries, they had to be alive, should have had no previous diagnosis of childhood solid tumour, and had to be living in the respective country at the time of diagnosis of the corresponding case. Birth order was obtained by identifying all maternal siblings in the population registries. We were able to obtain birth order for 3600 cases and 17 848 matching controls; in 43 cases, we were unable to identify the mother, and in Finland, we had information only on children born after 1986. Birth weight and gestational age were obtained from the Danish Medical Birth Registry (Gissler *et al*, 1997).

With regard to the analyses of variation of season of births, we included all children born between 1 January 1985 and 31 December 2006 in the Nordic countries who were registered in each of the national medical birth registries (Gissler *et al*, 1997). We restricted the analyses to cases born and diagnosed with a CNS tumour in the same period. Hence, this analysis included 2771 cases.

We were able to investigate the association between attendance at childcare in the first 2 years of life and risk of CNS tumours for a subset of children in Denmark. Information about childcare attendance was obtained from the Danish Childcare Database (Kamper-Jorgensen *et al*, 2007). We included children born between 1 January 1989 and 17 November 2002 and diagnosed with a CNS tumour during 1989–2006. To avoid confounding by indication, arising from childcare attendance being influenced by early symptoms of the tumour appearing in the first 2 years of life, we excluded children younger than 2 years of age at date of diagnosis. Of the 764 Danish children in whom CNS tumours were diagnosed in 1989–2006, 351 were eligible, and 3395 matched controls fulfilled the inclusion criteria. In these analyses, a 1 : 10 matching ratio was applied. For 3 cases and 15 controls, we were unable to trace information on municipality of residence.

In the analysis of birth order and childcare attendance, we used conditional logistic regression to estimate odds ratios (ORs) and 95% confidence intervals (CIs). Analyses were performed using the LOGISTIC procedure in SAS (version 9.1) (SAS institute Inc, Cary, NC, USA). Thus, by retaining the individual matching, we accounted for country, sex, and age in the analyses.

Walter and Elwood's test for a sinusoidal variation was applied to evaluate changes in CNS tumour incidence by month of birth, adjusted for the distribution of birth in the reference population (Walter and Elwood, 1975). The study was approved by the national data protection boards of all four countries and by ethical committees in accordance with national laws and regulations.

## Results

A primary CNS tumour was diagnosed in 3983 children in 1985–2006 (Table 1), with a small male preponderance. We found no association between birth order and CNS tumours overall or for most of the histological subgroups; all risk estimates were close to unity, with relatively narrow CIs (Table 2). However, a statistically significantly decreased risk for unspecified CNS tumours was observed among second-born children as opposed to first born (OR, 0.70; 95% CI, 0.51–0.95) (Table 2), but no protective effect of a higher birth order was found. Adjustment for gestational age and birth weight did not change the estimates markedly. Overall, no support of a seasonal variation of births was observed among the 2771 patients with a CNS tumour born and diagnosed in the period 1985–2006 (*P*=0.3) (Table 3). Further restriction of data to specific histological subgroups or age groups (0–4 and 5–14 years) did not show any seasonal variation that was different from that of the background population. Repeating the analyses by country did not show any seasonal variation for any tumour type in Denmark, Norway, and Sweden, but a statistically significant variation was found among children with ependymoma in Finland, with a peak of births in February; however, the numbers were small (63 Finnish children with ependymoma).

Information on childcare attendance during the first 2 years of life was complete for 170 cases and 1601 matched controls (Table 4). We found a small, but insignificantly increased OR for CNS tumours (OR, 1.29; 95% CI, 0.90–1.86) among children attending childcare during the first 2 years of life compared with those who stayed at home. Neither age at enrolment nor time enroled in childcare significantly affected the risk for CNS tumours. The OR was highest for children attending crèches (OR, 1.75; 95% CI, 1.04–2.93) and was lowest for childcare at home. In the analysis by histological subgroups, the OR for attendance at childcare was slightly higher for embryonal CNS tumours than for other brain tumours. This was especially the case for children enroled at younger ages, but is based on only 29 embryonal CNS tumour cases, and the CIs are wide.

We evaluated the effect of childcare in relation to sex, age at diagnosis, preterm birth, and birth order in restricted analysis (data not shown). No differences of the effects of attending childcare were observed between first born and later born children. In addition, we did not see any variation by age at diagnosis or sex. Restricting the data set to children born at term did not change the estimate (data not shown); however, few of the children in the study were born preterm.

## Discussion

In this study, the investigated proxy measures of infectious diseases were only weakly associated with CNS tumours in children, if at all. We found strong evidence that birth order has at the most a very small association with CNS tumours, consistent with most previous studies (Kuijten *et al*, 1990; McKinney *et al*, 1999; Schuz *et al*, 2001; Mogren *et al*, 2003; Von and Reynolds, 2003; Mallol-Mesnard *et al*, 2008). Birth order may not, however, be an accurate surrogate measure for all infections, as birth order affects the risk for infectious disease differently, depending on the virus or bacterium involved (Law, 2008).

Birth order may be a particularly poor proxy for exposure to childhood infections in Nordic countries, where a large proportion of children attend childcare (Kamper-Jorgensen *et al*, 2007), which is known to be an important source of contact with infectious agents. Furthermore, given the high prevalence of divorce and new family structures, birth order may not be a reliable measure of exposure to older children in the household.

We observed no seasonal variation of birth among children with any kind of CNS tumours in the Nordic countries. Hence, we did not confirm our previous observation of a seasonal variation of birth among children with ependymoma in Denmark (Schmidt *et al*, 2009), but the age groups and time period were not the same in the two studies. It is likely that the observed seasonal variations in subsets of the data are chance findings, even though we have observed a seasonal variation of birth among children with ependymoma, with a peak during winter, twice (previously in the Danish data and in the Finnish data of this study). The Walter and Edwood test might perform poorly when applied to too small sample sizes because of the fact that the asymptotic approximations that the test is based on are not valid for small numbers (St Leger, 1976; Roger, 1977), or it may simply be a result of multiple testing.

Seasonal variation in births of patients diagnosed with a specific disease, which differs from the underlying seasonality of births in the general population, suggests an environmental factor operating at conception, during pregnancy, in the neonatal period, or later in childhood. Such a seasonal pattern may reflect, for example, variation in exposure to sunlight or to infections. Most previous studies found no seasonal variation of birth among children with CNS tumour overall (Schmidt *et al*, 2009), although medulloblastoma has shown seasonality of birth in a number of studies, all with a peak during fall and winter (Yamakawa *et al*, 1979; Hoffman *et al*, 2007).

Our finding of a small nonsignificant increase in risk associated with childcare attendance should be interpreted carefully, as the numbers are small, resulting in wide CIs of the effect estimates. Furthermore, the lack of a dose–response relationship makes a biological phenomenon less likely. For embryonal CNS tumours, however, the direction of the estimates is more consistent, and this finding should be explored further in larger studies. In contrast to our findings, previous studies suggested a small protective effect of early social contact and childcare attendance (Shaw *et al*, 2006; Harding *et al*, 2009), but both studies were probably affected by recall and selection bias. Our use of the prospectively collected information from the Childcare Database minimised the risk for differential information bias, although a complete history of childcare attendance during the first 2 years of life was available for only 50% of the study participants. However, we have no reason to believe that this introduced selection bias, as the municipalities included in the database are representative of the Danish population. Confounding by indication is a potential problem, as children with nonspecific but unrecognised symptoms of CNS tumours may be less likely to be enroled in childcare at an early age. If present, this would result in an underestimation of the excess risk of childcare. In contrast, children with atopic disease might also be less likely to be enroled at childcare; as atopic disease is suggested to protect against CNS tumours (Linos *et al*, 2007; Harding *et al*, 2008), this would result in an overestimation of the effect.

The design and size of our study give high credibility to the results. The Nordic national health registries contain mandatory, continuously updated information on vital status, emigration status, birth, and cancer, and all patients have equal, free access to health care. Thus, use of these virtually complete population registries established for administrative purposes years before the current hypothesis was tested makes the probability of selection bias and information bias negligible.

Childhood CNS tumours comprise a heterogeneous group (Louis *et al*, 2007) of relatively rare tumours, each with their own characteristic genetic aberrations, clinical features, age-specific incidence rates and, possibly, aetiologies. The interpretation of studies on potential risk factors is further hampered by the use of different classifications, which affect overall as well as subgroup analyses. In this comprehensive Nordic population-based study, we had a substantial sample size to allow for analyses by histological subgroup.

Overall, the results do not support the hypothesis that exposure to infections in early childhood has a strong role in the pathogenesis of CNS tumours in children. However, we cannot rule out the possibility that a specific infectious agent is involved in the aetiology of CNS tumours.

## Figures and Tables

**Table 1:** Incident cases with CNS tumours by sex and histology, and overview of cases included in each analysis

	**Incident cases^b^**			**Birth order^c^**	**Seasonality^d^**	**Childcare^e^**
**Histology^a^**	**Boys**	**Girls**	**Total**	**Total**	**Total**	**Total**
Ependymoma	223	182	405	365	311	24
Astrocytoma	868	842	1710	1525	1128	128
PNET	412	280	692	648	519	49
Other gliomas	164	168	332	296	217	15
Other specified CNS tumours	304	237	541	497	378	37
Unspecified CNS tumours	163	140	303	269	218	65
						
Total	2134	1849	3983	3600	2771	348

Abbrevations: CNS=central nervous system; PNET=primitive neuroectodermal tumours.

a Classified according to the international classification of childhood cancer, 3rd edition.

b Incident cases of CNS tumours among children aged 0–14 years in Denmark, Norway, Finland, and Sweden in 1985–2006.

c Number of CNS tumour cases included in the analysis of birth order; in 43 cases, the mother could not be identified and in Finland, data was only available from 1987.

d Number of CNS tumour cases eligible for analysis of seasonal variation of birth. It includes patients born and diagnosed during 1985–2006.

e Number of cases eligible for analysis of childcare attendance. Born during 1985–2002 in Denmark and over 2 years of age.

**Table 2:** Birthorder and risk of CNS tumours in children by histological subgroup

	**Conditional OR**				**Conditional OR adjusted for birth weight and gestational age**			
**Histology/ birthorder**	**No. of cases**	**No. of controls**	**OR**	**95% CI**	**No. of cases**	**No. of controls**	**OR**	**95% CI**
*CNS tumours* a	3600	17 848			3337	16 299		
1	1571	7672	1.00	Ref.	1431	6849	1.00	Ref.
2	1277	6420	0.97	(0.90–1.05)	1210	5981	0.96	(0.88–1.05)
3	531	2650	0.97	(0.87–1.09)	493	2470	0.94	(0.84–1.06)
⩾4	221	1098	0.98	(0.84–1.15)	203	999	0.94	(0.80–1.11)
								
*Ependymoma*	365	1815			344	1678		
1	155	811	1.00	Ref.	141	726	1.00	Ref.
2	136	631	1.12	(0.87–1.45)	133	605	1.12	(0.86–1.45)
3	54	258	1.09	(0.77–1.53)	52	243	1.08	(0.76–1.53)
⩾4	20	115	0.91	(0.55–1.50)	18	104	0.86	(0.50–1.47)
								
*Astrocytoma*	1525	7547			1416	6933		
1	665	3214	1.00	Ref.	608	2898	1.00	Ref.
2	529	2759	0.93	(0.82–1.05)	501	2561	0.92	(0.81–1.05)
3	238	1112	1.03	(0.88–1.22)	224	1048	1.01	(0.85–1.20)
⩾4	93	426	0.97	(0.76–1.23)	83	426	0.88	(0.68–1.13)
								
*Embryonal CNS*	648	3221			613	2954		
1	284	1386	1.00	Ref.	268	1239	1.00	Ref.
2	237	1146	1.01	(0.84–1.22)	224	1081	0.96	(0.79–1.17)
3	86	476	0.88	(0.67–1.14)	83	438	0.85	(0.65–1.13)
⩾4	41	213	0.95	(0.66–1.35)	38	196	0.89	(0.62–1.30)
								
*Other glioma*	296	1463			266	1315		
1	121	632	1.00	Ref.	110	553	1.00	Ref.
2	109	512	1.11	(0.83–1.48)	100	471	1.03	(0.76–1.40)
3	50	231	1.13	(0.79–1.62)	41	212	0.95	(0.64–1.42)
⩾4	16	88	0.94	(0.53–1.67)	15	79	0.99	(0.91–1.07)
								
*Other specified tumours*	497	2460			451	2217		
1	214	1069	1.00	Ref.	185	941	1.00	Ref.
2	184	878	1.06	(0.85–1.31)	175	809	1.10	(0.87–1.39)
3	61	365	0.84	(0.62–1.14)	55	334	0.83	(0.59–1.15)
⩾4	38	148	1.28	(0.87–1.88)	36	133	1.35	(0.90–2.02)
								
*Unspecified tumours*	269	1342			247	1202		
1	132	560	1.00	Ref.	119	492	1.00	Ref.
2	82	494	0.70	(0.51–0.95)	77	454	0.70	(0.50–0.98)
3	42	216	0.83	(0.57–1.21)	38	195	0.81	(0.54–1.21)
⩾4	13	72	0.76	(0.40–1.41)	13	61	0.85	(0.45–1.63)

Abbrevations: 95% CI=95% confidence interval; CNS=central nervous system; No.=number; OR=odds ratio.

a Tumours classified according to the international classification of childhood cancer version 3, main group III are included.

**Table 3:** Seasonality of birth among children diagnosed with a CNS tumour in the Nordic countries

	**Age (years)**		**Sex**			**W&E's test^b^**	
**ICCC group** a	**0–4**	**5–14**	**Boys**	**Girls**	**Total**	***χ*^2^-value**	***P*-value**
Ependymoma	196	115	172	139	311	2.70	0.26
Astrocytoma	550	578	570	558	1128	3.35	0.19
Embryonal CNS tumour	283	236	306	213	519	0.79	0.67
Other glioma	82	135	103	114	217	0.49	0.78
Other specified CNS tumour	126	252	213	165	378	0.27	0.87
Unspecified CNS tumour	99	119	121	97	218	0.28	0.87
							
All CNS^c^	1336	1435	1485	1286	2771	2.35	0.31
							
*Analysis restricted by age group*							
All CNS aged 0–4 years						4.55	0.10
All CNS aged 5–14						0.45	0.80

Abbreviation: CNS=central nervous system.

a Tumours classified according to the international classification of childhood cancer (ICCC) version 3, main group III are included.

b Walther and Edwards test.

c In total 2771 children were born and diagnosed with a CNS tumour during 1985–2006.

Background population of the Nordic countries during 1985–2006 (10 575 793 children).

**Table 4:** Association with childcare attendance and CNS tumours overall, astrocytoma, and embryonal CNS tumour

	**All CNS tumours^a^**				**Astrocytoma**				**Embryonal CNS tumour**			
	**No. of cases**	**No. of controls**	**OR^b^**	**95% CI**	**No. of cases**	**No. of controls**	**OR^b^**	**95% CI**	**No. of cases**	**No. of controls**	**OR^b^**	**95% CI**
Children in analysis	348	3380			128	1258			49	488		
												
*Attendance to childcare*												
Ever	125	1090	1.29	(0.90–1.86)	46	390	1.19	(0.67–2.13)	23	194	1.47	(0.57–3.77)
Never	45	511	1.00	Ref.	19	188	1.00	Ref.	6	73	1.00	Ref.
Incomplete registration^c^	178	1779	1.12	(0.78–1.61)	63	680	0.84	(0.47–1.51)	20	221	1.05	(0.40–2.80)
												
*Age at enrolment in childcare*					128	1258			49	488		
Never	45	511	1.00	Ref.	19	188	1.00	Ref.	6	73	1.00	Ref.
<6 months	21	185	1.29	(0.75–2.24)	6	57	1.06	(0.41–2.79)	8	30	3.39	(1.08–10.71)
6–11 months	53	491	1.20	(0.79–1.85)	21	174	1.23	(0.62–2.42)	9	86	1.32	(0.44–3.92)
12–17 months	34	288	1.32	(0.82–2.13)	13	114	1.15	(0.53–2.46)	6	53	1.36	(0.42–4.41)
18–23 months	17	126	1.55	(0.86–2.81)	6	45	1.34	(0.51–3.54)	0	25	d	
Incomplete registration^c^	178	1779	1.12	(0.78–1.61)	63	680	0.84	(0.47–1.51)	20	221	1.04	(0.39–2.76)
												
*Time enroled in childcare*					128	1258			49	488		
Never	45	511	1.00	Ref.	19	188	1.00	Ref.	6	73	1.00	Ref.
<6 months	20	140	1.63	(0.93–2.85)	6	48	1.25	(0.48–3.29)	0	29	d	
6–11 months	34	301	1.27	(0.79–2.04)	13	123	1.05	(0.49–2.25)	7	54	1.55	(0.49–4.84)
12–23 months	71	649	1.23	(0.82–1.83)	27	219	1.26	(0.66–2.39)	16	111	1.84	(0.68–4.97)
Incomplete registration^c^	178	1779	1.12	(0.78–1.61)	63	680	0.84	(0.47–1.51)	20	221	1.04	(0.39–2.76)
												
*Type of daycare* e												
Never	45	511	1.00	Ref.	19	188	1.00	Ref.	6	73	1.00	ref
Crèche only	26	168	1.75	(1.04–2.93)	8	61	1.36	(0.56–3.30)	5	28	2.25	(0.63–8.05)
Daycare home only	65	138	1.15	(0.77–1.73)	25	229	1.11	(0.59–2.12)	11	114	1.18	(0.41–3.33)
Age-integrated only	22	174	1.39	(0.81–2.40)	7	65	1.07	(0.43–2.67)	4	29	1.71	(0.45–6.51)
Enroled in various types	12	110	1.26	(0.78–1.61)	6	35	1.82	(0.64–5.15)	3	23	1.65	(0.36–7.48)
Incomplete registration^c^	178	1779	1.12	(0.78–1.61)	63	680	0.84	(0.47–1.51)	20	221	1.04	(0.39–2.77)

Abbrevations: 95%CI=95% confidence interval; CN=central nervous system; No.=number; OR=odds ratio.

a Tumours classified according to the international classification of childhood cancer version 3, main group III are included.

b Adjusted for matching variables, namely, sex, month, and year of birth.

c Includes children for whom the complete history of childcare during the first 2 years was not known, for example, moving to an uncovered municipality, migration, or death.

d No cases with embryonal CNS tumour were in this category.

e Daycare homes enrol up to five children aged 0–3 years, crèches enrol an average of 40 children aged 0–3 years, and age-integrated facilities enrol a mean of 73 children aged 0–6 years.
